# Supplementary material for: Cryo-EM structures of human Cx36/GJD2 neuronal gap junction channel
Source: Nat Commun. 2023 Mar 11;14:1347. doi: 10.1038/s41467-023-37040-8 (PMC10008584; doi:10.1038/s41467-023-37040-8)
Supplement: Supplementary file 1 — Supplementary Information [file 41467_2023_37040_MOESM1_ESM.pdf]

Supplementary Information

Cryo-EM structures of human Cx36/GJD2 neuronal gap junction channel

Seu-Na Lee, Hwa-Jin Cho, Hyeongseop Jeong, Bumhan Ryu, Hyuk-Joon Lee, Minsoo Kim, Jejoong Yoo, Jae-Sung Woo, Hyung Ho Lee

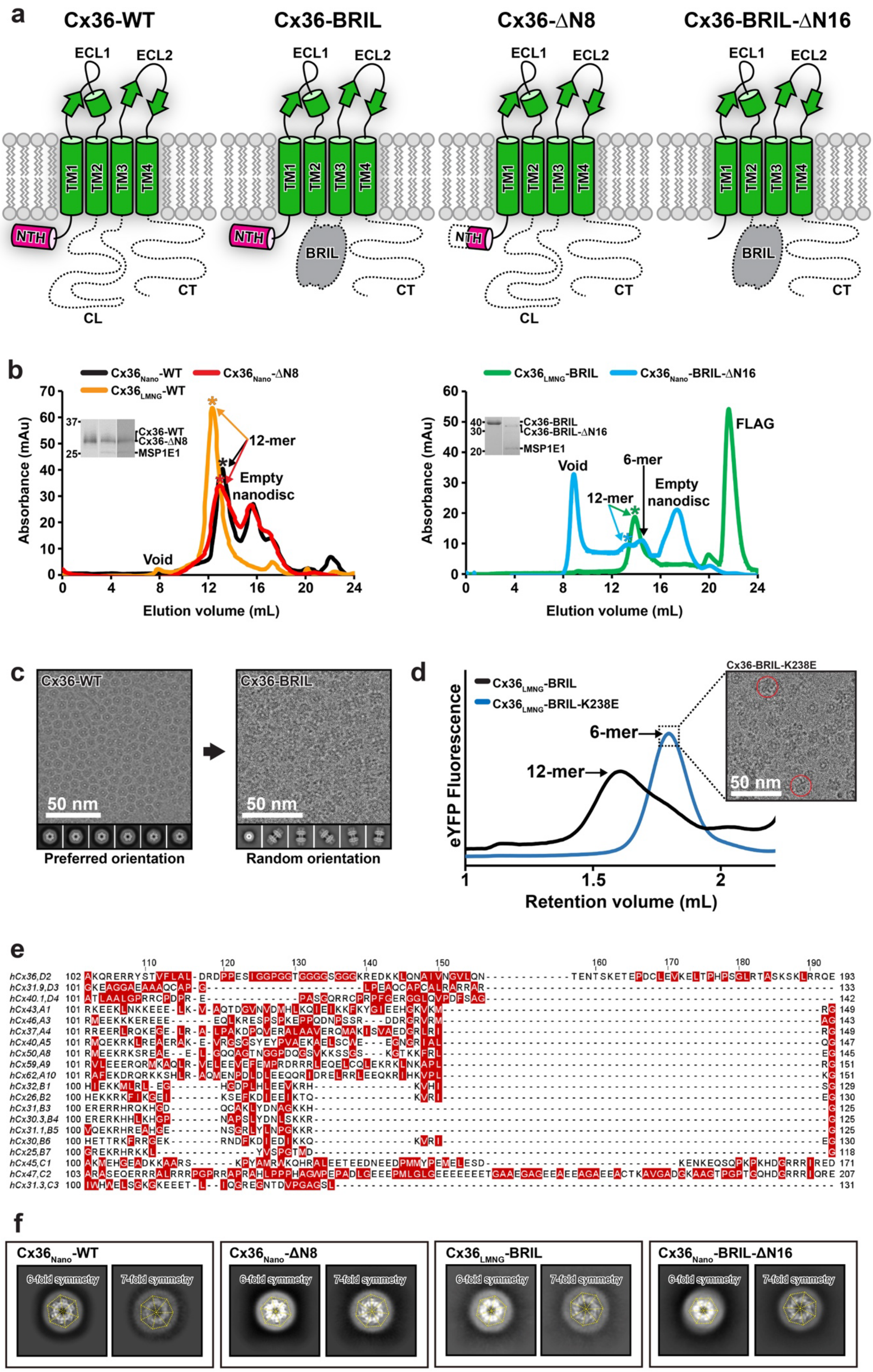
a
Cx36-WT
Cx36-BRIL
Cx36-ΔN8
Cx36-BRIL-ΔN16
ECL1
ECL2
TM1
TM2
TM3
TM4
NTH
BRIL
CL
CT
b
Cx36Nano-WT
Cx36Nano-ΔN8
Cx36LMNG-WT
Cx36-WT
Cx36-ΔN8
MSP1E1
12-mer
Empty nanodisc
Void
Absorbance (mAu)
Elution volume (mL)
Cx36LMNG-BRIL
Cx36Nano-BRIL-ΔN16
Cx36-BRIL
Cx36-BRIL-ΔN16
6-mer
FLAG
c
50 nm
Preferred orientation
Random orientation
d
Cx36LMNG-BRIL
Cx36LMNG-BRIL-K238E
Cx36-BRIL-K238E
eYFP Fluorescence
Retention volume (mL)
e
hCx36,D2
hCx31.9,D3
hCx40.1,D4
hCx43,A1
hCx46,A3
hCx37,A4
hCx40,A5
hCx50,A8
hCx59,A9
hCx62,A10
hCx32,B1
hCx26,B2
hCx31,B3
hCx30.3,B4
hCx31.1,B5
hCx30,B6
hCx25,B7
hCx45,C1
hCx47,C2
hCx31.3,C3
f
Cx36Nano-WT
Cx36Nano-ΔN8
Cx36LMNG-BRIL
Cx36Nano-BRIL-ΔN16
6-fold symmetry
7-fold symmetry

Supplementary Fig. 1: Construct design, purification, and cryo-EM imaging of Cx36 GJC. **a** Schematic representation of the Cx36 constructs used in this study. NTH is colored magenta, and the deleted region of NTH (residues 2-8) is shown as an empty cylinder. The α-helices and β-sheets are represented as green cylinders and arrows, respectively. The flexible CLs and CTs are shown as dashed lines. BRILs are colored gray and outlined by dashed lines. **b** Size-exclusion chromatography and SDS-PAGE results of $Cx36_{Nano}$-WT (black), $Cx36_{Nano}$-ΔN8 (red), $Cx36_{LMNG}$-WT (orange), $Cx36_{LMNG}$-BRIL (green), and $Cx36_{Nano}$-BRIL-ΔN16 (sky blue). The SDS-PAGE results of purified $Cx36_{LMNG}$-WT (left), $Cx36_{Nano}$-WT (middle), and $Cx36_{Nano}$-ΔN8 (right) were shown in left panel, and those of $Cx36_{LMNG}$-BRIL (left) and $Cx36_{Nano}$-BRIL-ΔN16 (right) are in right panel. **c** Micrographs and representative 2D classes of $Cx36_{LMNG}$-WT (left) and $Cx36_{LMNG}$-BRIL (right). The side-view population was largely increased by the replacement of CL (residues 109-187) with BRIL. **d** Fluorescence-detection size exclusion chromatography of $Cx36_{LMNG}$-BRIL (black) and $Cx36_{LMNG}$-BRIL-K238E (blue), and cryo-EM micrographs of $Cx36_{LMNG}$-BRIL-K238E. Some side-views of $Cx36_{LMNG}$-BRIL-K238E hemichannels are marked by red circles. **e** Amino acid sequence alignment of the CL regions in all connexin families except the highly diversified Cx23. The hydrophobic residues are shaded in red. Cx36 has the second longest CL (residues 102-193) in the connexin family, which is highly hydrophobic. **f** Identification of GJCs or hemichannels with 7-fold symmetry for all Cx36 constructs used in this study. Representative 2D classes of top-views with 6-fold (left panels) and 7-fold (right panels) symmetries are presented for each indicated Cx36 construct. Abbreviations: NTH, N-terminal helix; TM, transmembrane helix; ECL, extracellular loop; CL, cytoplasmic loop; CT, C-terminal tail; MSP1E1, membrane scaffold protein 1 E1. The purification and cryo-EM experiments in Supplementary Fig. 1b-d were repeated more than three times with similar results. Source data for size-exclusion

chromatography and fluorescence-detection size exclusion chromatography are provided as a Source Data file.

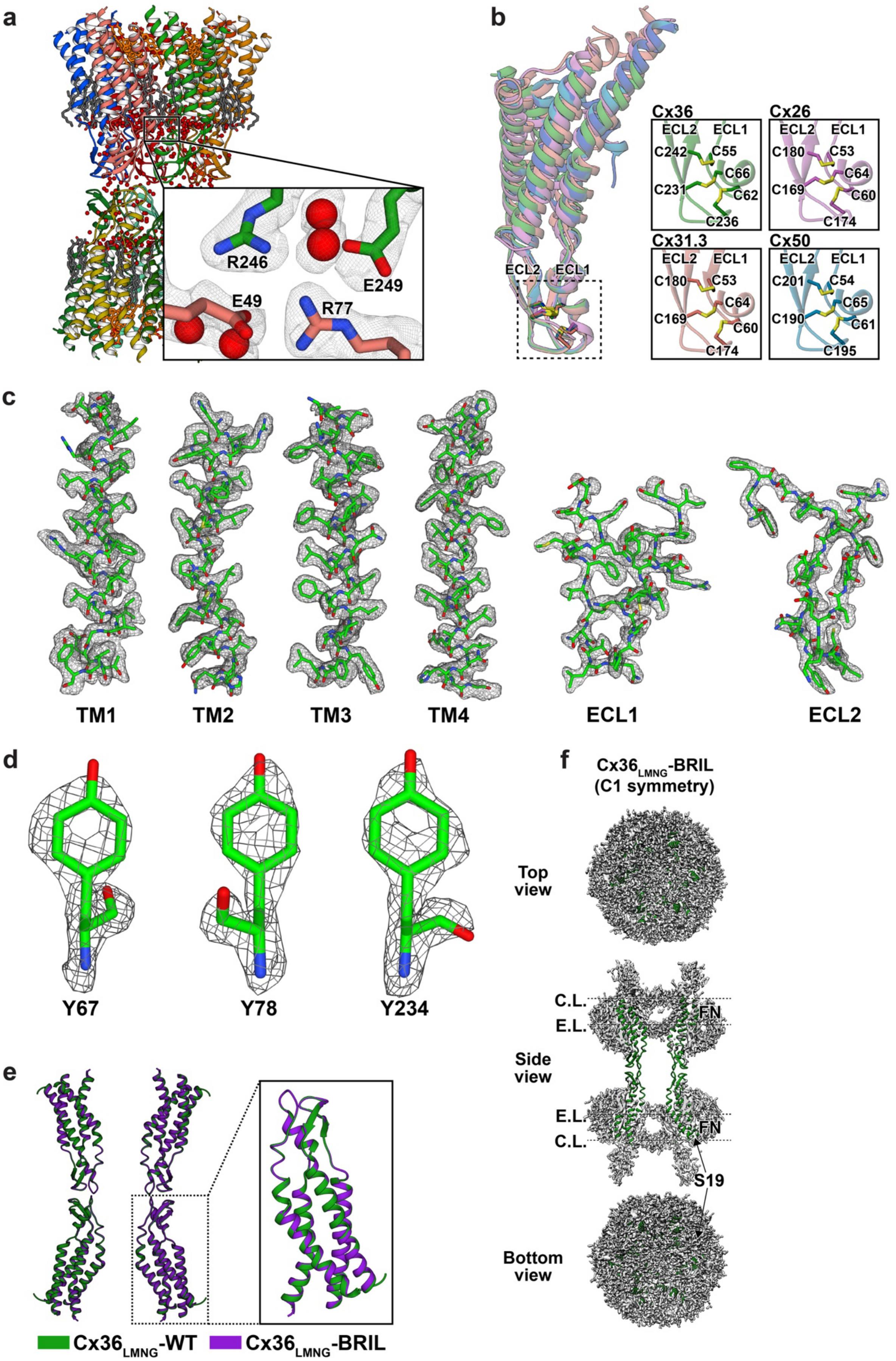

a
R246
E249
E49
R77
b
ECL2
ECL1
Cx36
ECL2 ECL1
C242 C55
C66
C231 C62
C236
Cx26
ECL2 ECL1
C180 C53
C64
C169 C60
C174
Cx31.3
ECL2 ECL1
C180 C53
C64
C169 C60
C174
Cx50
ECL2 ECL1
C201 C54
C65
C190 C61
C195
c
TM1
TM2
TM3
TM4
ECL1
ECL2
d
Y67
Y78
Y234
e
Cx36$_{LMNG}$-WT
Cx36$_{LMNG}$-BRIL
f
Cx36$_{LMNG}$-BRIL (C1 symmetry)
Top view
Side view
C.L.
E.L.
FN
E.L.
FN
C.L.
S19
Bottom view

Supplementary Fig. 2: Detailed structures of $Cx36_{LMNG}$-BRIL GJC. a Water molecules bound to the channel are shown as red spheres. The solvent tunnel between neighboring protomers and between TMD and ECLs are blocked by four charged residues (Glu49, Arg77, Arg246, and Glu249). In the close-up view, map densities of water molecules and the four residues are represented as meshes. **b** Superposition of Cx36, Cx26, Cx31.3, and Cx50 structures. The three disulfide bonds are strictly conserved in all structures. The accession codes for Cx26, Cx31.3, and Cx50 are 2ZW3, 6L3T, and 7JJP, respectively. **c** Map densities of TM1 (residues 19-46), TM2 (residues 75-101), TM3 (residues 194-220), TM4 (residues 247-276), ECL1 (residues 47-74), and ECL2 (residues 221-246). **d** Representative aromatic ring densities are shown for Tyr67, Tyr78, and Tyr234. **e** Superimposition of $Cx36_{LMNG}$-WT (green) and $Cx36_{LMNG}$-BRIL (purple) structures. The RMSD was 0.55 Å. **f** Top, cross-sectioned side, and bottom views of the 3D map reconstructed with C1 symmetry imposition. Ribbon representation of the FN state colored in green. The densities of detergent micelles, pore-occluding molecules and BRIL are displayed as white densities. C.L. and E.L. denote cytoplasmic layer and extracellular layer, respectively.

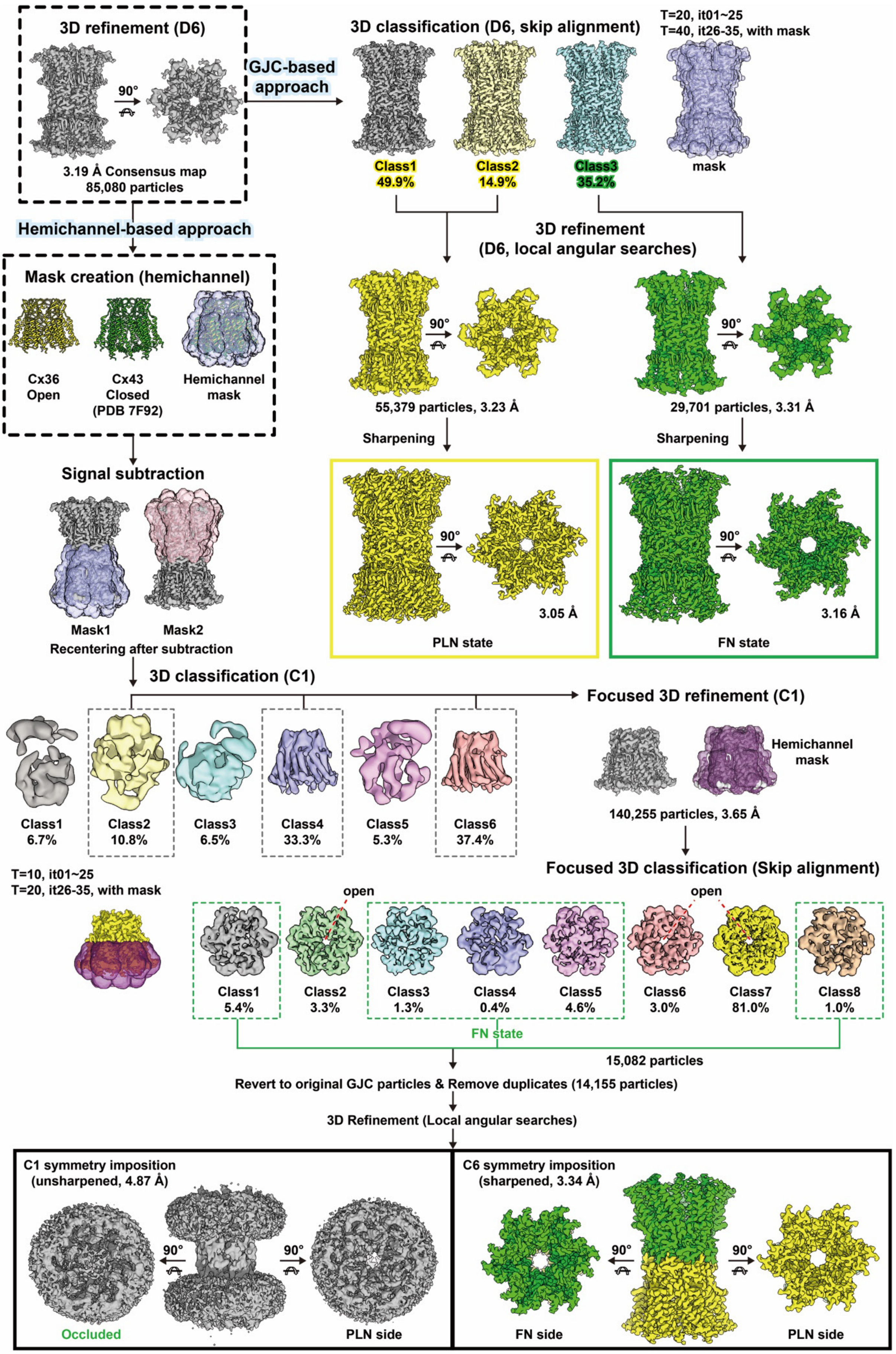

3D refinement (D6)
90°
3.19 Å Consensus map
85,080 particles
GJC-based approach
3D classification (D6, skip alignment)
T=20, it01~25
T=40, it26-35, with mask
Class1 49.9%
Class2 14.9%
Class3 35.2%
mask
Hemichannel-based approach
3D refinement
(D6, local angular searches)
Mask creation (hemichannel)
Cx36 Open
Cx43 Closed (PDB 7F92)
Hemichannel mask
55,379 particles, 3.23 Å
29,701 particles, 3.31 Å
Sharpening
Sharpening
Signal subtraction
3.05 Å
3.16 Å
Mask1
Mask2
Recentering after subtraction
PLN state
FN state
3D classification (C1)
Focused 3D refinement (C1)
Hemichannel mask
Class1 6.7%
Class2 10.8%
Class3 6.5%
Class4 33.3%
Class5 5.3%
Class6 37.4%
140,255 particles, 3.65 Å
Focused 3D classification (Skip alignment)
T=10, it01~25
T=20, it26-35, with mask
open
open
Class1 5.4%
Class2 3.3%
Class3 1.3%
Class4 0.4%
Class5 4.6%
Class6 3.0%
Class7 81.0%
Class8 1.0%
FN state
15,082 particles
Revert to original GJC particles & Remove duplicates (14,155 particles)
3D Refinement (Local angular searches)
C1 symmetry imposition
(unsharpened, 4.87 Å)
C6 symmetry imposition
(sharpened, 3.34 Å)
Occluded
PLN side
FN side
PLN side

Supplementary Fig. 3: Workflow of cryo-EM image processing and structure determination of $Cx36_{Nano}$-WT GJC. The full PLN and full FN conformations were identified by the GJC-focused 3D classification. The structurally hetero-junctional GJC was reconstructed using the full FN hemichannel classes in the hemichannel-focused 3D classification. See Methods for details.

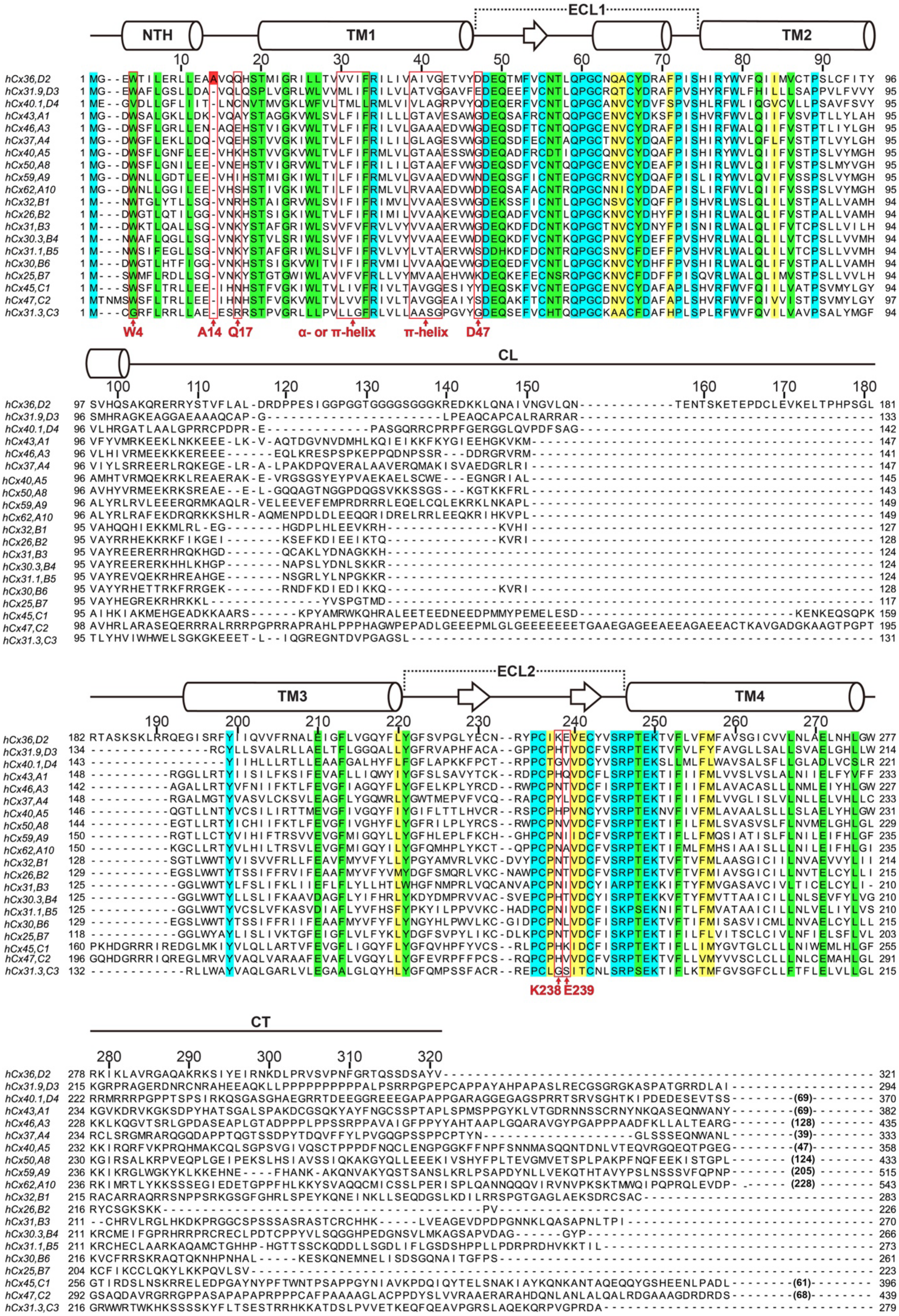

ECL1
NTH
TM1
TM2
W4
A14
Q17
α- or π-helix
π-helix
D47
CL
ECL2
TM3
TM4
K238
E239
CT
hCx36,D2
hCx31.9,D3
hCx40.1,D4
hCx43,A1
hCx46,A3
hCx37,A4
hCx40,A5
hCx50,A8
hCx59,A9
hCx62,A10
hCx32,B1
hCx26,B2
hCx31,B3
hCx30.3,B4
hCx31.1,B5
hCx30,B6
hCx25,B7
hCx45,C1
hCx47,C2
hCx31.3,C3

Supplementary Fig. 4: Amino acid sequence alignment of human connexins. All human connexins except highly diversified Cx23 were included in the sequence alignment. The conserved residues are highlighted by yellow (80%), green (90%), or cyan (100%) colors. Ala14 is shaded by red. Trp4, Ala14, Gln17, residues 30-33 (α- or π-helix), residues 39-42 (π-helix), Asp47, K238, and E239 in Cx36 are indicated by red boxes and labeled. Schematic drawings of the secondary structures in Cx36 are shown above the amino acid sequence. Coils, α-helices, and β-sheets are represented as solid lines, cylinders, and arrows, respectively.

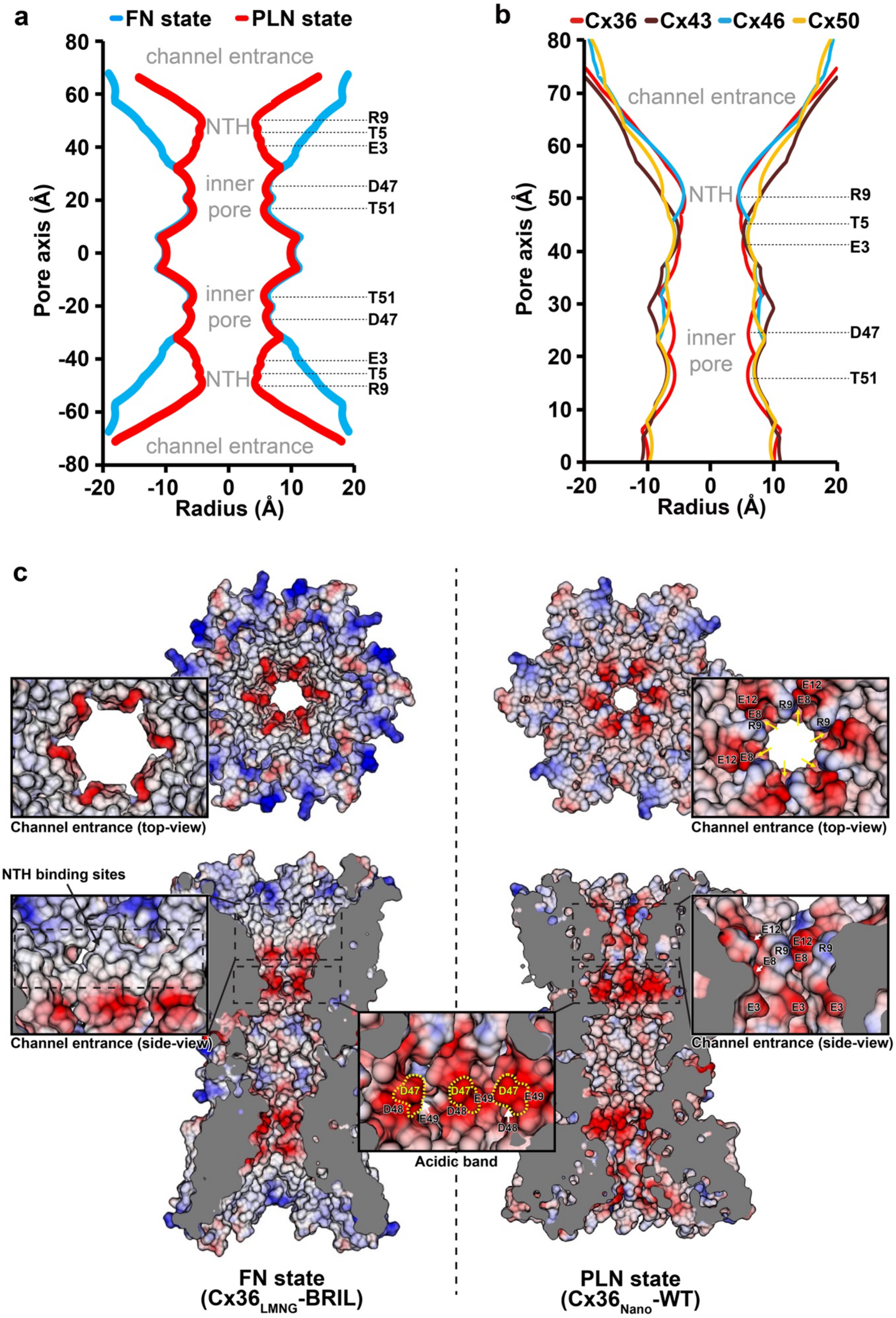

a
FN state
PLN state
channel entrance
NTH
inner pore
R9
T5
E3
D47
T51
Pore axis (Å)
Radius (Å)
b
Cx36
Cx43
Cx46
Cx50
channel entrance
NTH
inner pore
R9
T5
E3
D47
T51
Pore axis (Å)
Radius (Å)
c
Channel entrance (top-view)
NTH binding sites
Channel entrance (side-view)
Acidic band
D47
D48
E49
E12
E8
R9
E3
FN state
(Cx36$_{LMNG}$-BRIL)
PLN state
(Cx36$_{Nano}$-WT)

Supplementary Fig. 5: Comparison of pore properties between the FN and PLN states of Cx36 GJC and between available GJC structures. a Comparison of solvent-accessible pore diameters between the FN ($Cx36_{LMNG}$-BRIL, blue line) and PLN states ($Cx36_{Nano}$-WT, red line) of Cx36 GJC. **b** Comparison between the pore diameters of Cx36 (red line), Cx43 (brown line), Cx46 (skyblue line), and Cx50 (orange line). **c** Surface electrostatic potentials in FN and PLN states of Cx36 GJC. Top views and cross-sectioned side views of the FN ($Cx36_{LMNG}$-BRIL) and PLN ($Cx36_{Nano}$-WT) states, respectively. The surface potential ranges from -5 (red) to +5 (blue) kT/e. Asp47, Asp48, and Glu49 in the acidic band are labeled. Asp47 is highlighted by yellow color. The solvent-accessible pore diameters were calculated using HOLE program[1]. Source data for solvent-accessible pore diameters are provided as a Source Data file.

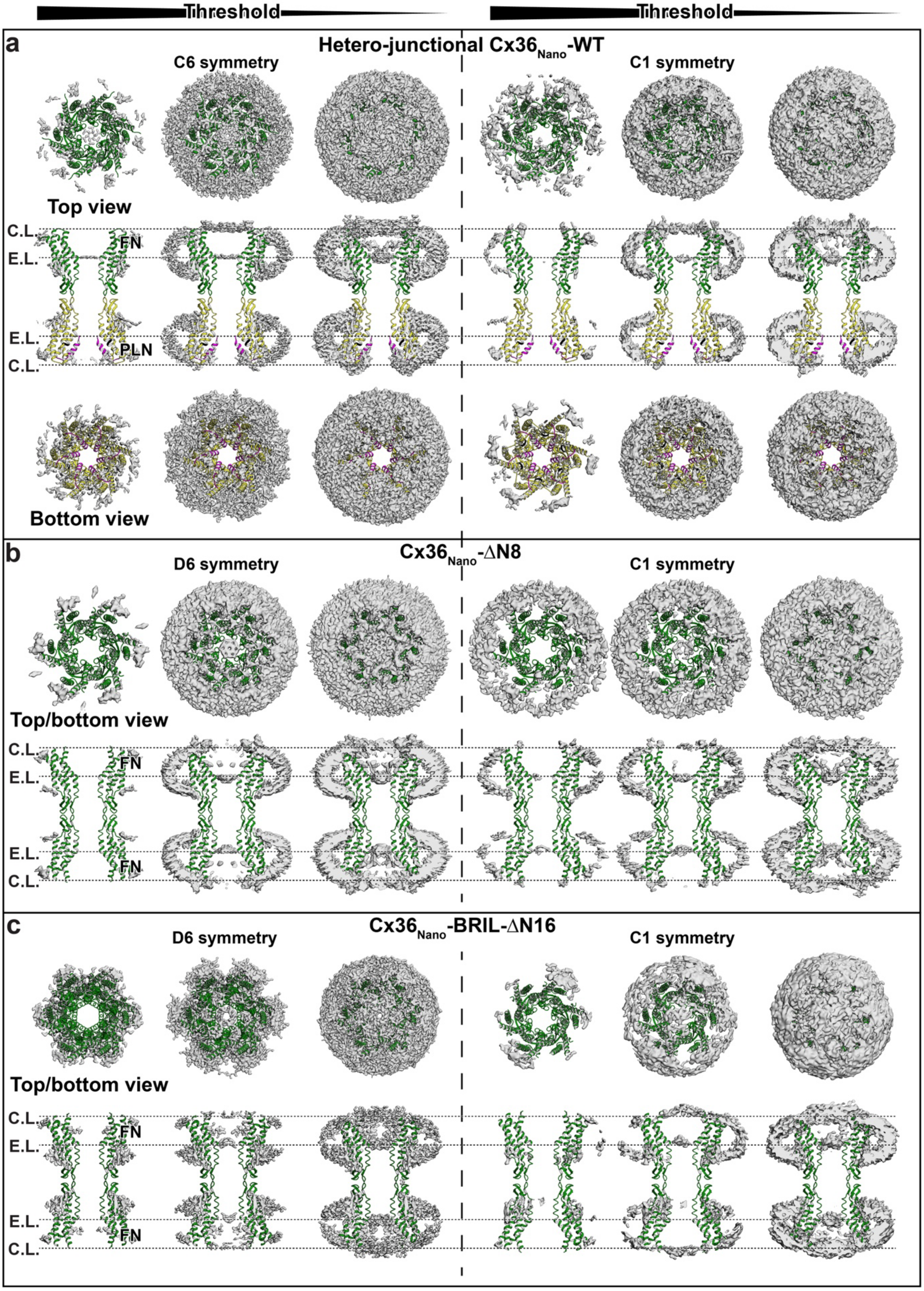

Threshold
Threshold
a
Hetero-junctional Cx36$_{Nano}$-WT
C6 symmetry
C1 symmetry
Top view
C.L.
E.L.
FN
E.L.
PLN
C.L.
Bottom view
b
Cx36$_{Nano}$-ΔN8
D6 symmetry
C1 symmetry
Top/bottom view
C.L.
FN
E.L.
E.L.
FN
C.L.
c
Cx36$_{Nano}$-BRIL-ΔN16
D6 symmetry
C1 symmetry
Top/bottom view
C.L.
FN
E.L.
E.L.
FN
C.L.

Supplementary Fig. 6: Pore-occluding densities in $Cx36_{Nano}$-WT, $Cx36_{Nano}$-ΔN8, and $Cx36_{Nano}$-BRIL-ΔN16 GJCs. a-c Top view, cross-sectioned side, and bottom views of cryo-EM reconstruction map with the indicated symmetry imposition are shown at three different map contour levels. Lipids and nanodiscs densities are colored in gray. The ribbon drawings of Cx36 GJCs in the FN and PLN states are colored in green and yellow, respectively. NTH and π-helix (residues 30-33) are highlighted by magenta and black colors, respectively. C.L. and E.L. denote cytoplasmic layer and extracellular layer, respectively.

MD simulation of Cx36 in PLN state with CL

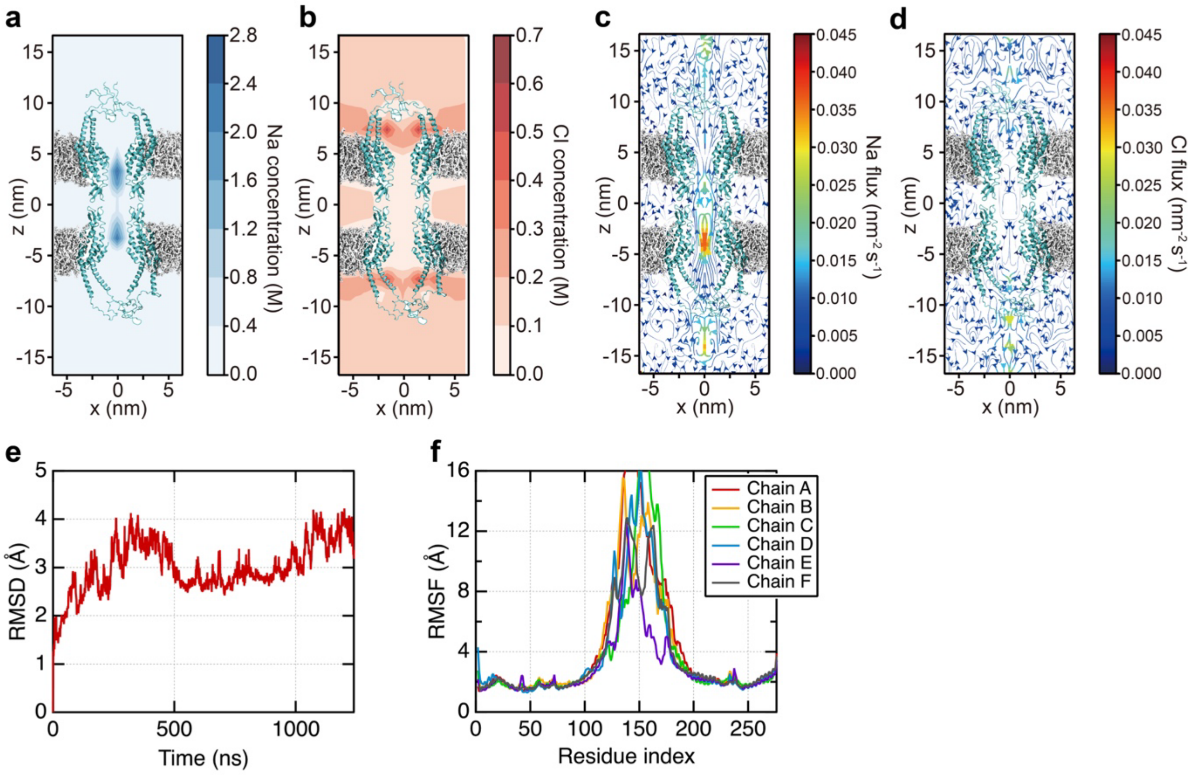

MD simulation of Cx36 in PLN state without CL

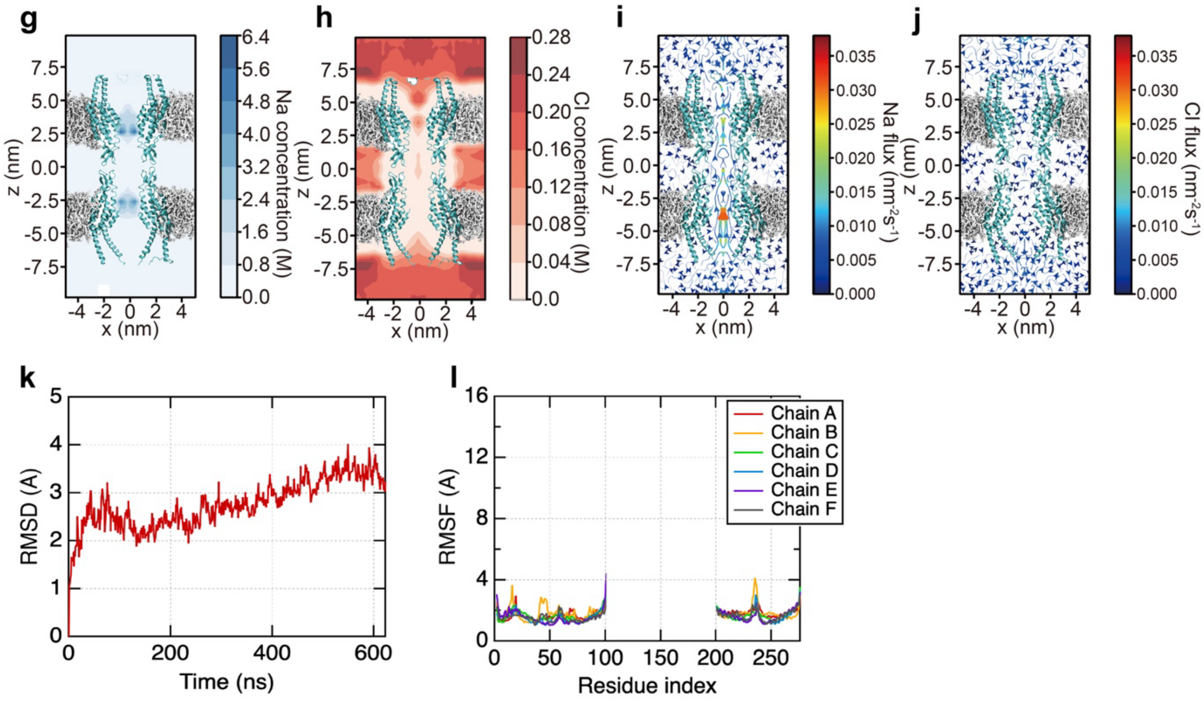

Supplementary Fig. 7: Molecular dynamics simulation of Cx36 GJC in the full PLN conformation. The MD simulation is performed using the structural model of $Cx36_{Nano}$-WT GJC in the full PLN state with (a-f) and without a predicted CL model (g-l). **a-b, g-h** Local concentration maps of Na^{+} (a and g) and Cl^{-} (b and h) were computed without transjunctional potential. **c-d, i-j** Ionic fluxes of Na^{+} (c and i) and Cl^{-} (d and j) under a trans-junctional potential of 200 mV. **e-f, k-l** The root mean square deviation (RMSD) of Cx36 GJC during the indicated trajectories and the root mean square fluctuation (RMSF) of each protomer in the Cx36 hemichannel region.

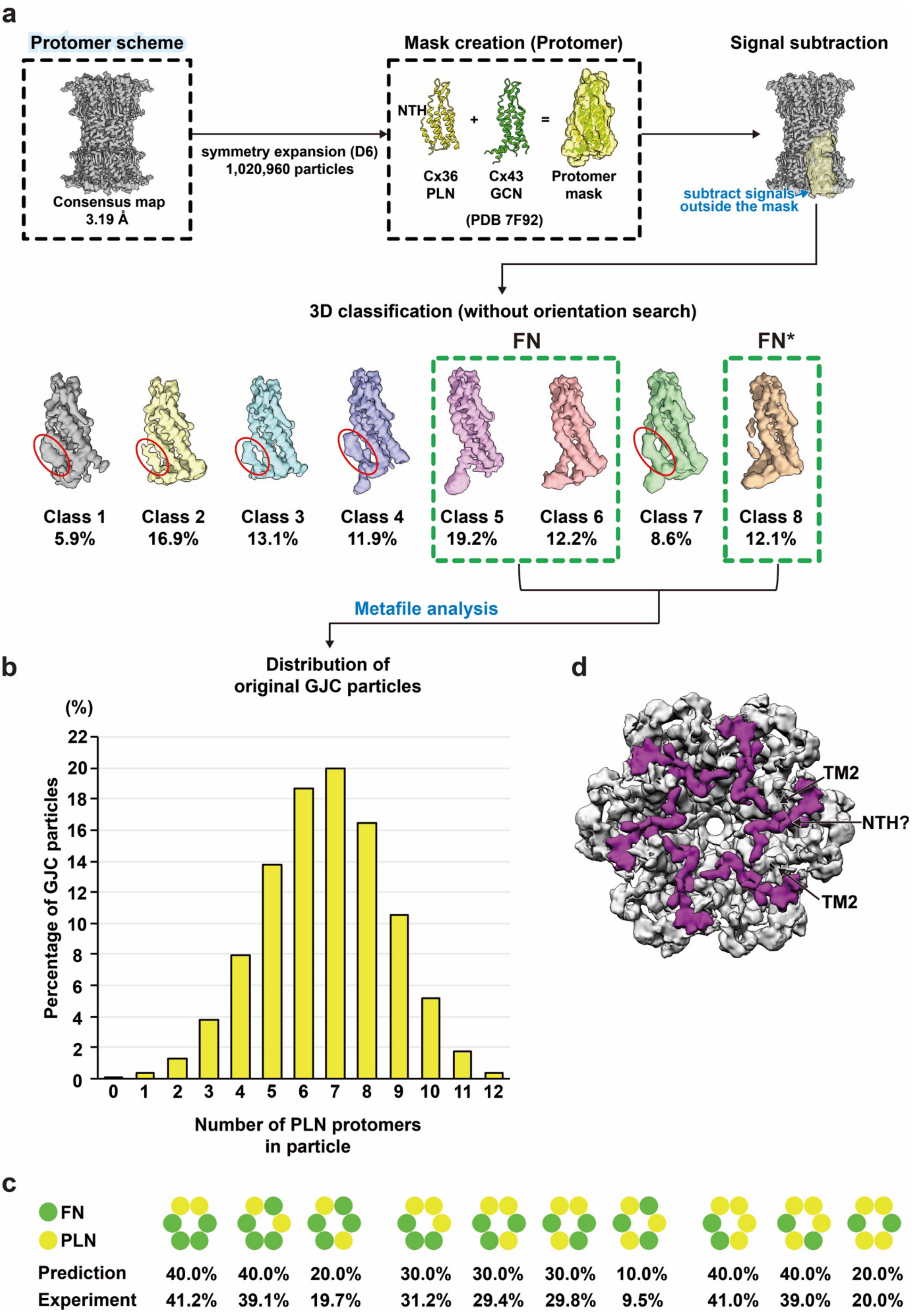
a
Protomer scheme
Consensus map
3.19 Å
symmetry expansion (D6)
1,020,960 particles
Mask creation (Protomer)
NTH
Cx36
PLN
Cx43
GCN
Protomer
mask
(PDB 7F92)
Signal subtraction
subtract signals
outside the mask
3D classification (without orientation search)
FN
FN*
Class 1
5.9%
Class 2
16.9%
Class 3
13.1%
Class 4
11.9%
Class 5
19.2%
Class 6
12.2%
Class 7
8.6%
Class 8
12.1%
Metafile analysis
b
Distribution of
original GJC particles
(%)
Percentage of GJC particles
Number of PLN protomers
in particle
d
TM2
NTH?
TM2
c
FN
PLN
Prediction
40.0%
40.0%
20.0%
30.0%
30.0%
30.0%
10.0%
40.0%
40.0%
20.0%
Experiment
41.2%
39.1%
19.7%
31.2%
29.4%
29.8%
9.5%
41.0%
39.0%
20.0%

Supplementary Fig. 8: Protomer-focused 3D classification of $Cx36_{Nano}$-WT GJC. a Workflow of protomer-based conformational variation analysis using the $Cx36_{Nano}$-WT dataset. See the Methods section for detailed description. The densities of NTHs are indicated by red circles to easily identify the PLN classes. Green dotted-line boxes indicate the FN classes. The asterisk indicates an FN class (class 8) with the map density possibly corresponding to GCN. **b** Distribution of the number of PLN protomers in each GJC particle. **c** Positional distribution of PLN protomers in each hemichannel region. Three groups of the hemichannel regions each containing two, three, and four PLN protomers were separately analyzed. The experimental data were compared with the predicted percentages of the indicated distributions when PLN protomers were randomly distributed. **d** Structure of the hemichannel region of $Cx36_{Nano}$-WT GJC that might be partly in the GCN conformation. Map densities possibly corresponding to NTHs are colored in magenta.

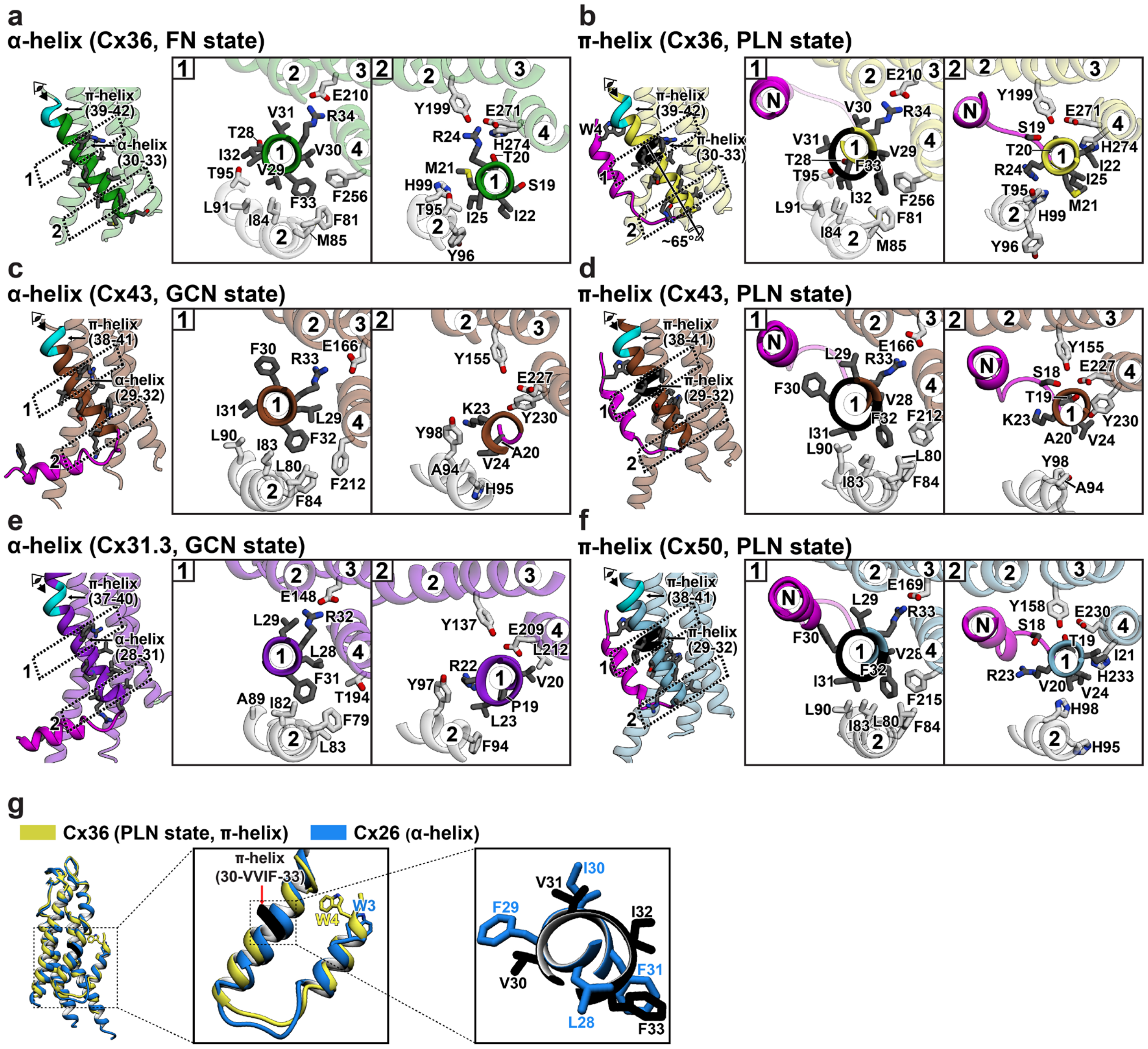

Supplementary Fig. 9: Detailed structural changes in Cx36 and comparison with other connexin structures. a-f Structural comparison of $Cx36_{Nano}$-WT in the FN (a) and PLN (b) states, Cx43 in the GCN (c) and PLN states (d), Cx31.3 in GCN state (e), and Cx50 in PLN state (f). NTH, π-helix in residues 30-33, and π-helices in residues 39-42 of Cx36 (a, b) are colored in magenta, black, and cyan, respectively, and the corresponding regions of Cx43 (c, d), Cx31.3 (e), and Cx50 (f) are represented in the same colors. During the transition from the FN to the PLN state of Cx36, the cytoplasmic half (residues 20-33) of TM1 undergoes ~65° helical rotation (b). Each connexin structure was cross-sectioned at two levels indicated by dotted lines (cross-sections 1 and 2) and viewed from the extracellular side. Dramatic changes

in the interaction network of TM helices are caused by the α-to-π-helix transition in TM1. **g** Superposition of $Cx36_{Nano}$-WT in the PLN state (yellow) and Cx26 (PDB ID: 2ZW3, blue) with RMSD of 2.26 Å. While residues 30-33 of Cx36 form π-helix (black), the corresponding residues of Cx26 (residues 28-31) show α-helix.

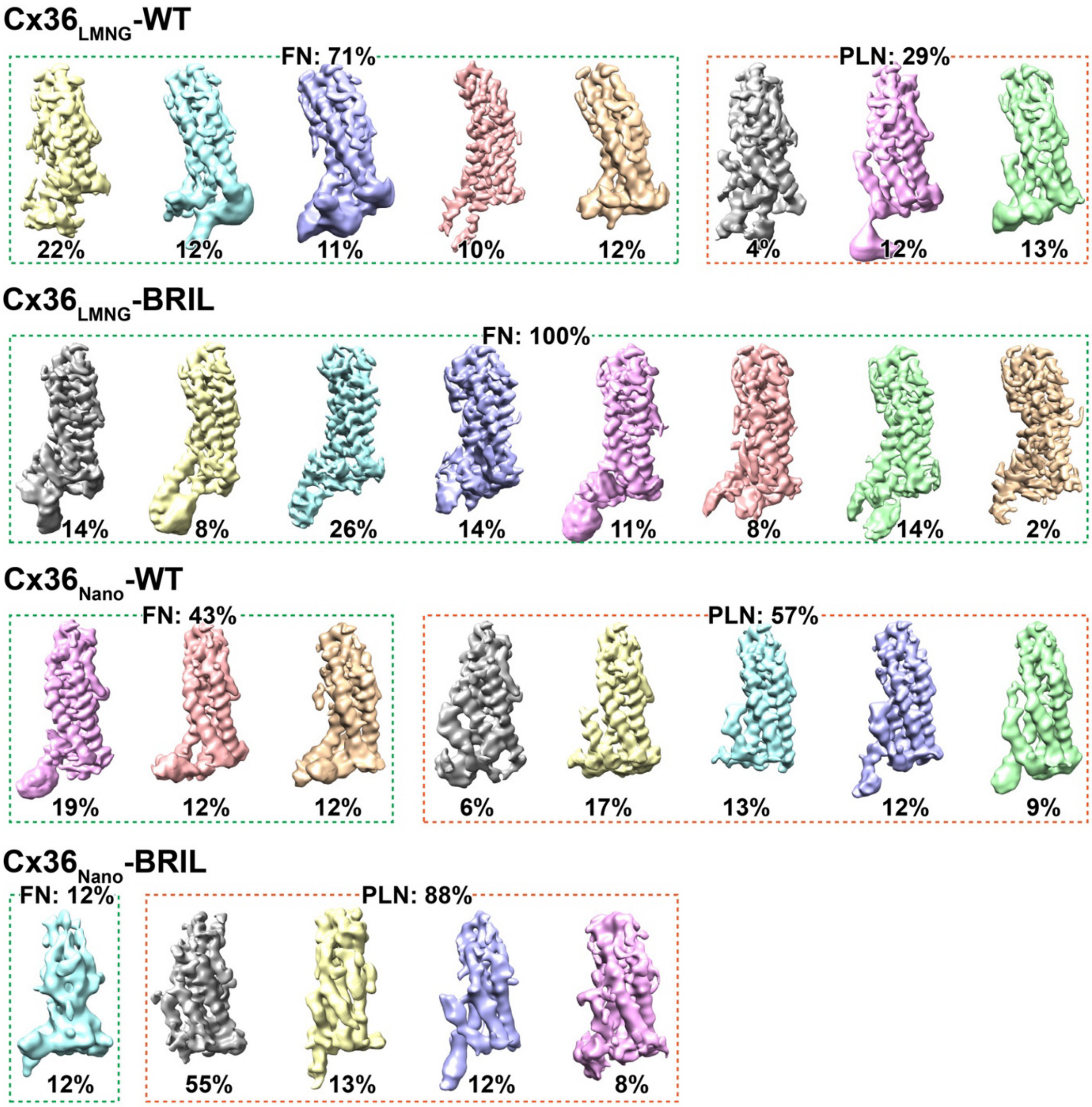

Supplementary Fig. 10: Protomer-focused 3D classification of $Cx36_{LMNG}$-WT, $Cx36_{LMNG}$-BRIL, $Cx36_{Nano}$-WT and $Cx36_{Nano}$-BRIL. All protomer classes that do not show clear map density of pore-lining NTH were classified as the FN conformation.

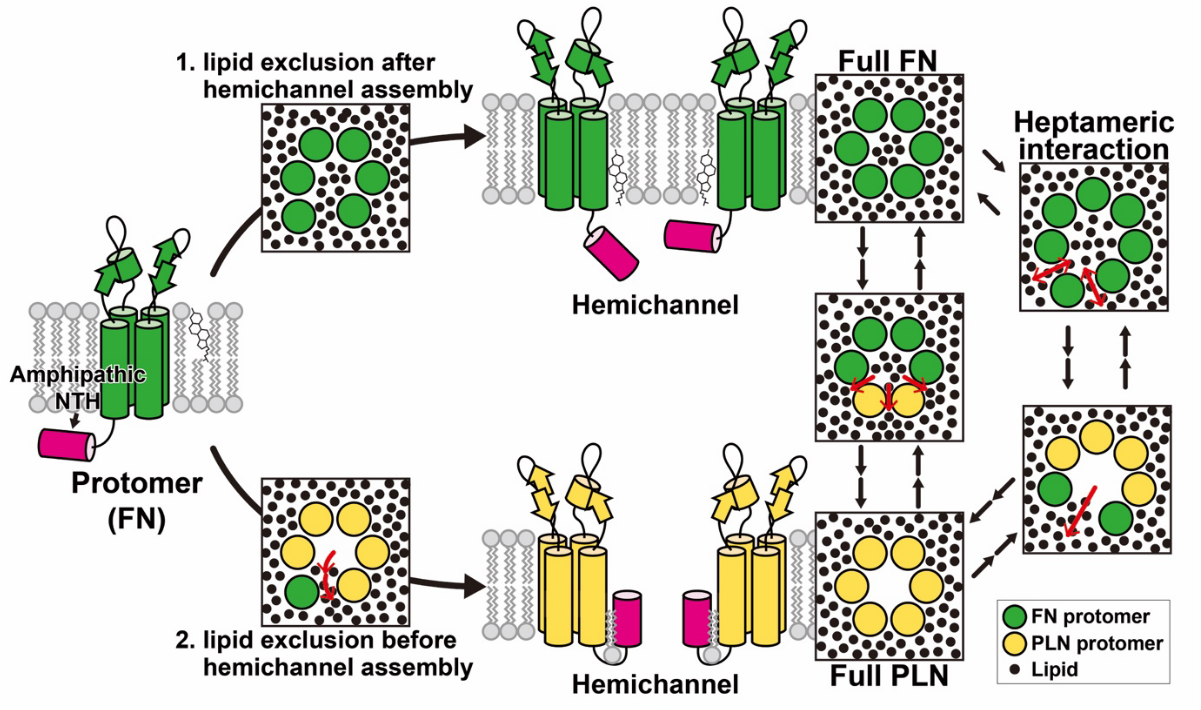

Supplementary Fig. 11: Possible scenarios of lipid exclusion during the channel assembly process. A hypothetical model of stepwise lipid exclusion after the completion of channel assembly is compared with an unlikely model of immediate lipid exclusion before the completion of channel assembly. The α-helices and β-sheets are represented as cylinders and arrows, respectively. The amphipathic NTH is represented as a magenta cylinder. In the boxes that display the snapshots of the channel assembly process viewed from the top, FN protomers, PLN protomers, and membrane lipids are represented as green, yellow, and black circles. Red arrows indicate movement of lipids.

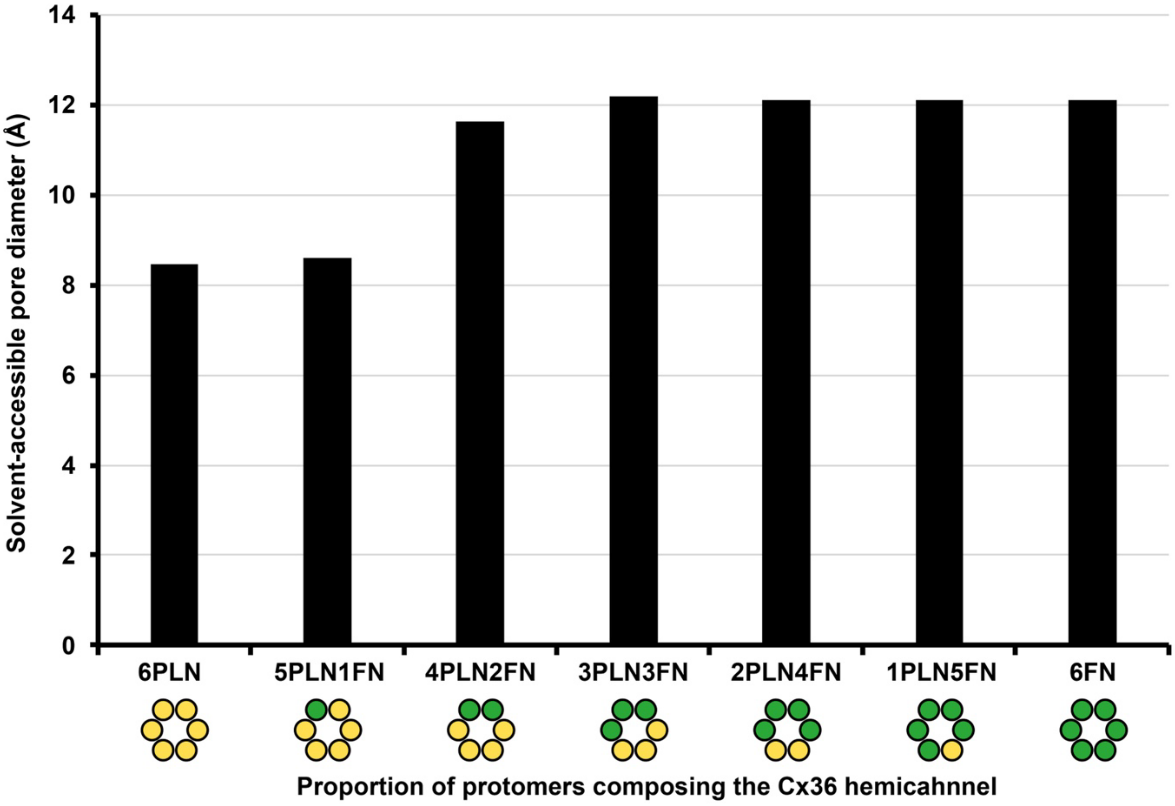

Supplementary Fig. 12: Pore sizes of the Cx36 hemichannel region in different compositions of PLN and FN. Each hemichannel region with a mixture of PLN and FN (4:2 to 0:6 ratios) shows a pore with a solvent accessible diameter of ~12 Å, which is much larger than that of the full PLN GJC (8.5 Å). The solvent-accessible pore diameters were calculated using HOLE program[1]. Yellow and green circles indicate PLN and FN protomers, respectively. Source data for solvent-accessible pore diameters are provided as a Source Data file.

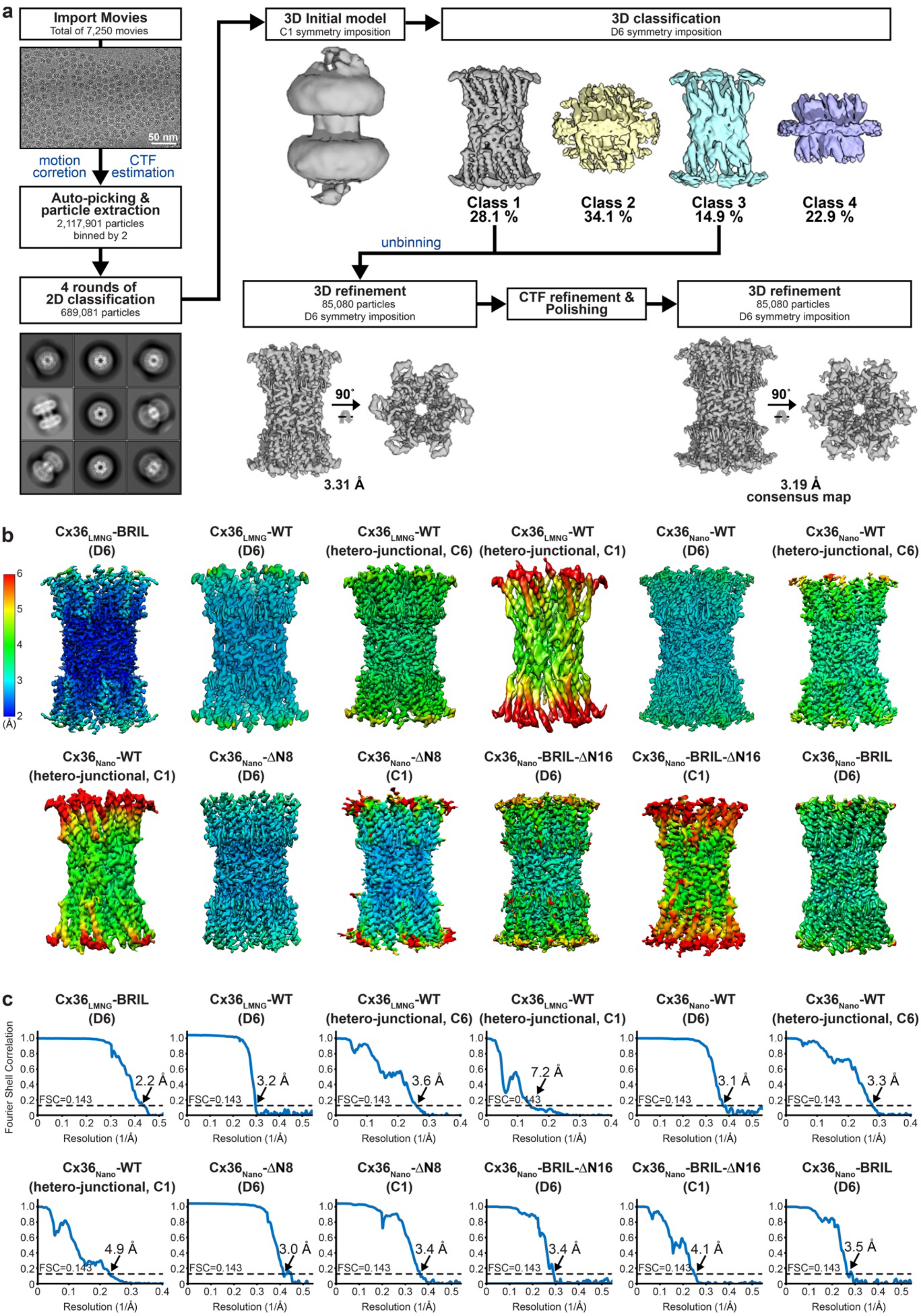
a
Import Movies
Total of 7,250 movies
50 nm
motion corretion
CTF estimation
Auto-picking & particle extraction
2,117,901 particles
binned by 2
4 rounds of 2D classification
689,081 particles
3D Initial model
C1 symmetry imposition
3D classification
D6 symmetry imposition
Class 1
28.1 %
Class 2
34.1 %
Class 3
14.9 %
Class 4
22.9 %
unbinning
3D refinement
85,080 particles
D6 symmetry imposition
CTF refinement & Polishing
3D refinement
85,080 particles
D6 symmetry imposition
90°
3.31 Å
90°
3.19 Å
consensus map
b
Cx36LMNG-BRIL (D6)
Cx36LMNG-WT (D6)
Cx36LMNG-WT (hetero-junctional, C6)
Cx36LMNG-WT (hetero-junctional, C1)
Cx36Nano-WT (D6)
Cx36Nano-WT (hetero-junctional, C6)
6
5
4
3
2
(Å)
Cx36Nano-WT (hetero-junctional, C1)
Cx36Nano-ΔN8 (D6)
Cx36Nano-ΔN8 (C1)
Cx36Nano-BRIL-ΔN16 (D6)
Cx36Nano-BRIL-ΔN16 (C1)
Cx36Nano-BRIL (D6)
c
Cx36LMNG-BRIL (D6)
Cx36LMNG-WT (D6)
Cx36LMNG-WT (hetero-junctional, C6)
Cx36LMNG-WT (hetero-junctional, C1)
Cx36Nano-WT (D6)
Cx36Nano-WT (hetero-junctional, C6)
Fourier Shell Correlation
2.2 Å
3.2 Å
3.6 Å
7.2 Å
3.1 Å
3.3 Å
FSC=0.143
Resolution (1/Å)
Cx36Nano-WT (hetero-junctional, C1)
Cx36Nano-ΔN8 (D6)
Cx36Nano-ΔN8 (C1)
Cx36Nano-BRIL-ΔN16 (D6)
Cx36Nano-BRIL-ΔN16 (C1)
Cx36Nano-BRIL (D6)
4.9 Å
3.0 Å
3.4 Å
3.4 Å
4.1 Å
3.5 Å
FSC=0.143
Resolution (1/Å)

Supplementary Fig. 13: A flow chart of cryo-EM image processing and data validation. a Flow chart illustrating the cryo-EM image processing procedure of $Cx36_{Nano}$-WT (see Methods). This method was also applied to other cryo-EM data sets used in this study. **b-c** Local resolution and Fourier shell correlations (c) of twelve Cx36 maps reconstructed in this study. The local resolution was estimated using ResMap. The local resolution ranges from 2 Å (blue) to 6 Å (red). The estimated resolution was 2.2-7.2 Å (c). Source data for Fourier shell correlations are provided as a Source Data file.

Supplementary Table 1. Summarization of cryo-EM structures determined in this study.

Structure (Map resolution)	PDB ID & EMDB ID	Expression	Environment	Conformation (symmetry)	Protein engineering
$Cx36_{LMNG}$-WT (3.2 Å)	7XKK & EMD-33256	HEK293	Detergents	FN state (D6)	-
$Cx36_{LMNG}$-WT (3.6 Å)	8HKP & EMD-34856	HEK293	Detergents	Hetero-junctional GJC (C6)	-
$Cx36_{LMNG}$-WT (7.2 Å)	EMD-34857	HEK293	Detergents	Hetero-junctional GJC (C1)	-
$Cx36_{Nano}$-WT (3.1 Å)	7XNH & EMD-33315	HEK293	Nanodiscs	PLN state (D6)	-
$Cx36_{Nano}$-WT (3.3 Å)	7XNV & EMD-33327	HEK293	Nanodiscs	Hetero-junctional GJC (C6)	-
$Cx36_{Nano}$-WT (4.9 Å)	N/A & EMD-33328	HEK293	Nanodiscs	Hetero-junctional GJC (C1)	-
$Cx36_{Nano}$-ΔN8 (3.0 Å)	7XL8 & EMD-33274	HEK293	Nanodiscs	FN state (D6)	Deletion: residues 2-8
$Cx36_{Nano}$-ΔN8 (3.4 Å)	N/A & EMD-33275	HEK293	Nanodiscs	FN state (C1)	Deletion: residues 2-8
$Cx36_{LMNG}$-BRIL (2.2 Å)	7XKT & EMD-33270	Sf9	Detergents	FN state (D6)	Substitution: CL (residues 109-188) to BRIL (residues 21-128)
$Cx36_{Nano}$-BRIL (3.4 Å)	EMD-34822	Sf9	Nanodiscs	PLN state (D6)	Substitution: CL (residues 109-188) to BRIL (residues 21-128)
$Cx36_{Nano}$-BRIL-ΔN16 (3.4 Å)	7XKI & EMD-33254	Sf9	Nanodiscs	FN state (D6)	Substitution: CL (residues 109-188) to BRIL (residues 21-128) Deletion: residues 2-16
$Cx36_{Nano}$-BRIL-ΔN16 (4.5 Å)	N/A & EMD-33255	Sf9	Nanodiscs	FN state (C1)	Substitution: CL (residues 109-188) to BRIL (residues 21-128) Deletion: residues 2-16

Supplementary Table 2. Cryo-EM data collection, refinement, and validation statistics

	**Cx36-WT**								**. Cx36-BRIL**			
	$Cx36_{LMNG}$-WT in detergents	$Cx36_{LMNG}$-WT (hetero-junctional) in detergents	$Cx36_{LMNG}$-WT (hetero-junctional) in detergents	$Cx36_{Nano}$-WT (PLN) in nanodiscs	$Cx36_{Nano}$-WT (hetero-junctional) in nanodiscs	$Cx36_{Nano}$-WT (hetero-junctional) in nanodiscs	$Cx36_{Nano}$-ΔN8 in nanodiscs	$Cx36_{Nano}$-ΔN8 in nanodiscs	$Cx36_{LMNG}$-BRIL in detergents	$Cx36_{Nano}$-BRIL in nanodiscs	$Cx36_{Nano}$-BRIL-ΔN16 in nanodiscs	$Cx36_{Nano}$-BRIL-ΔN16 in nanodiscs
	EMD-33256 PDB 7XKK	EMD-34856 PDB 8HKP	EMD-34857 N/A	EMD-33315 PDB 7XNH	EMD-33327 PDB 7XNV	EMD-33328 N/A	EMD-33274 PDB 7XL8	EMD-33275 N/A	EMD-33270 PDB 7XKT	EMD-34822 N/A	EMD-33254 PDB 7XKI	EMD-33255 N/A
**Data collection and processing**												
Magnification	96,000	96,000	96,000	130,000	130,000	130,000	105,000	105,000	96,000	96,000	96,000	96,000
Voltage (kV)	300	300	300	300	300	300	300	300	300	300	300	300
Electron exposure (e–/Å^2)	40	40	40	49	49	49	61	61	40	40	40	40
Defocus range (μm)	-1.5 – -2.75	-1.5 – -2.75	-1.5 – -2.75	-1.5 – -2.75	-1.5 – -2.75	-1.5 – -2.75	-0.9 – -2.4	-0.9 – -2.4	-1.5 – -3.0	-1.5 – -2.75	-1.5 – -3.0	-1.5 – -3.0
Pixel size (Å)	0.675	0.675	0.675	0.658	0.658	0.658	0.8415	0.8415	0.675	0.675	0.675	0.675
Symmetry imposed	D6	C6	C1	D6	C6	C1	D6	C1	D6	D6	D6	C1
Initial particle images (no.)	609,292	609,292	609,292	689,081	689,081	689,081	766,823	766,823	435,817	71,238	426,090	426,090
Final particle images (no.)	103,180	103,180	103,108	55,379	14,155	14,155	460,806	460,806	70,095	10,611	39,444	39,444
Map resolution (Å)	3.2	3.6	7.2	3.1	3.3’	4.9	3.0	3.41	2.2	3.4	3.4	4.5
FSC threshold												
**Refinement**												
Initial model used (PDB code)	7XKT	7XKT			7XNH, 7XKT		7XKT				7XKT	
Model resolution (Å)	3.4	3.5		3.0	3.4		3.2		2.4		3.2	
FSC threshold												
Map sharpening *B* factor (Å^2)	170.7	108.3		-127	-91.9		128.4	89.9	68.5	71.2	-125	107.2
Model composition												
Non-hydrogen atoms	16,044	17,256		19,224	18,378		16,920		18,480		16,284	
Protein residues	1,980	2088		2,172	2,088		1,956		1,992		1,992	
Ligands		AV0: 6		MC3: 132	MC3: 114		MC3: 84		MC3: 72<br>LHG: 24<br>Y01:24		MC3: 48	
B factors (Å^2)												
Protein	30.54	31.88		22.05	25.93		60.52		48.29		61.49	
Ligand				37.75	38.80		51.20		55.02		33.68	
R.m.s. deviations												
Bond lengths (Å)	0.006	0.005		0.004	0.004		0.009		0.019		0.002	
Bond angles (°)	0.820	0.642		0.637	0.677		1.060		1.737		0.444	
Validation												
MolProbity score	1.25	1.36		1.36	1.41		1.70		1.04		0.97	
Clashscore	3.62	4.55		6.61	7.45		15.56		2.52		1.34	
Poor rotamers (%)	1.33	0.00		0.00	0.90		0.68		0.67		0.00	
Ramachandran plot												
Favored (%)	99.38	97.30		99.44	98.38		98.74		99.13		97.48	
Allowed (%)	0.62	2.70		0.00	1.03		1.26		0.87		2.52	
Disallowed (%)	0.00	0.00		0.56	0.59		0.00		0.00		0.00	

Supplementary Table 3. Summarization of primers used in this study.

	DNA	Sequence (5'-3')
Cx36-WT	Cx36-WT_F	5'-GCGCTGTCGACATGGGCGAGTGGACGATCTT-3'
	Cx36-WT_R	5'-TCTAGAGACGTACGCGCTGTCTGAGG-3'
$Cx36_{Nano}$-ΔN8	Cx36-ΔN8_F	5'-CGGTTGCTGGAGGCAGCGGTTCAGCAGCATAGC-3'
	Cx36-ΔN8_R	5'-GCTGCCTCCAGCAACCGCATGTCGACCTGTCAG-3'
Cx36-BRIL	Cx36-BRIL_Primer1_F	5'-CTCTTAAGGGATCCGCCACCATGGGGGAATGGACCATCTT-3'
	Cx36-BRIL_Primer1_R	5'-CAATTGTCTTCAAGATCAGCGCGGCGTTCTCGCTGCTTGG-3'
	Cx36-BRIL_Primer2_F	5'-CCTATATCCAGAAGTATCTGAAGCTCAGAAGGCAGGAAGG-3'
	Cx36-BRIL_Primer2_R	5'-AGCTTGTCGAGACTGCAGGCCTACTTATCGTCGTCATCCT-3'
	BRIL(23-128)_F	5'-GCTGATCTTGAAGACAATTG-3'
	BRIL(23-128)_R	5'-CAGATACTTCTGGATATAGG-3'
Cx36-BRIL-ΔN16	Cx36-BRIL-ΔN16_Primer1_F	5'-TTAAGGGATCCGCCACCATGCAGCACTCCACTATGATCGG-3'
	Cx36-BRIL-ΔN16_Primer1_R	5'-AGCTTGTCGAGACTGCAGGCCTACTTATCGTCGTCATCCT-3'

Supplementary Reference

1 Smart, O. S. *et al*. HOLE: a program for the analysis of the pore dimensions of ion channel structural models. *J. Mol. Graph.* **14.6**, 354-360 (1996).
