# Supplementary material for: Cryo-EM structures of human Cx36/GJD2 neuronal gap junction channel
Source: Nat Commun. 2023 Mar 11;14:1347. doi: 10.1038/s41467-023-37040-8 (PMC10008584; doi:10.1038/s41467-023-37040-8)
Supplement: Supplementary file 3 — Reporting Summary [file 41467_2023_37040_MOESM3_ESM.pdf]

nature portfolio

Corresponding author(s): Jae-Sung Woo
Hyung Ho Lee

Last updated by author(s): Jan 25, 2023

Reporting Summary

Nature Portfolio wishes to improve the reproducibility of the work that we publish. This form provides structure for consistency and transparency in reporting. For further information on Nature Portfolio policies, see our Editorial Policies and the Editorial Policy Checklist.

Statistics

For all statistical analyses, confirm that the following items are present in the figure legend, table legend, main text, or Methods section.

n/a	Confirmed	
☐	☒	The exact sample size (*n*) for each experimental group/condition, given as a discrete number and unit of measurement
☐	☒	A statement on whether measurements were taken from distinct samples or whether the same sample was measured repeatedly
☒	☐	The statistical test(s) used AND whether they are one- or two-sided<br>*Only common tests should be described solely by name; describe more complex techniques in the Methods section.*
☒	☐	A description of all covariates tested
☐	☒	A description of any assumptions or corrections, such as tests of normality and adjustment for multiple comparisons
☐	☒	A full description of the statistical parameters including central tendency (e.g. means) or other basic estimates (e.g. regression coefficient) AND variation (e.g. standard deviation) or associated estimates of uncertainty (e.g. confidence intervals)
☒	☐	For null hypothesis testing, the test statistic (e.g. *F*, *t*, *r*) with confidence intervals, effect sizes, degrees of freedom and *P* value noted<br>*Give P values as exact values whenever suitable.*
☐	☒	For Bayesian analysis, information on the choice of priors and Markov chain Monte Carlo settings
☐	☒	For hierarchical and complex designs, identification of the appropriate level for tests and full reporting of outcomes
☒	☐	Estimates of effect sizes (e.g. Cohen's *d*, Pearson's *r*), indicating how they were calculated

Our web collection on statistics for biologists contains articles on many of the points above.

Software and code

Policy information about availability of computer code

Data collection	Cryo-EM data collection was performed using EPU 2.10.0.5REL.
Data analysis	In this study, following softwares were used: cryoSPARC 3.1.0, Relion 3.1, PyEM, Coot 0.9.6, Phenix 1.19, UCSF Chimera 1.16, UCSF ChimeraX 1.4, GROMACS package 2022.1, LINCS algorithm, SETTLE algorithm.

For manuscripts utilizing custom algorithms or software that are central to the research but not yet described in published literature, software must be made available to editors and reviewers. We strongly encourage code deposition in a community repository (e.g. GitHub). See the Nature Portfolio guidelines for submitting code & software for further information.

Data

Policy information about availability of data

All manuscripts must include a data availability statement. This statement should provide the following information, where applicable:

- Accession codes, unique identifiers, or web links for publicly available datasets
- A description of any restrictions on data availability
- For clinical datasets or third party data, please ensure that the statement adheres to our policy

Twelve cryo-EM density maps for Cx36LMNG-BRIL, Cx36Nano-BRIL, Cx36LMNG-WT, structurally hetero-junctional Cx36LMNG-WT (C6 and C1 symmetry imposition), Cx36Nano-WT in PLN state, structurally hetero-junctional Cx36Nano-WT (C6 and C1 symmetry imposition), Cx36Nano-ΔN8 (D6 and C1 symmetry imposition) and Cx36Nano-BRIL-ΔN16 (D6 and C1 symmetry imposition) have been deposited at the Electron Microscopy Data Bank (EMDB) under accession codes EMD-33270,

EMD-34822, EMD-33256, EMD-34856, EMD-34857, EMD-33315, EMD-33327, EMD-33328, EMD-33274, EMD-33275, EMD-33254 and EMD-33255, respectively. Seven coordinates for the model of Cx36LMNG-BRIL, Cx36LMNG-WT, structurally hetero-junctional Cx36LMNG-WT (C6 symmetry imposition), Cx36Nano-WT in PLN state, structurally hetero-junctional Cx36Nano-WT (C6 symmetry imposition), Cx36Nano-ΔN8 (D6 symmetry imposition), and Cx36Nano-BRIL-ΔN16 (D6 symmetry imposition) have been deposited at the RCSB Protein Data Bank (PDB) under accession codes 7XKT, 7XKK, 8HKP, 7XNH, 7XNV, 7XL8 and 7XKI, respectively.

Human research participants

Policy information about studies involving human research participants and Sex and Gender in Research.

Reporting on sex and gender	N/A
Population characteristics	N/A
Recruitment	N/A
Ethics oversight	N/A

Note that full information on the approval of the study protocol must also be provided in the manuscript.

Field-specific reporting

Please select the one below that is the best fit for your research. If you are not sure, read the appropriate sections before making your selection.

☒ Life sciences ☐ Behavioural & social sciences ☐ Ecological, evolutionary & environmental sciences

For a reference copy of the document with all sections, see nature.com/documents/nr-reporting-summary-flat.pdf

Life sciences study design

All studies must disclose on these points even when the disclosure is negative.

Sample size	For cryo-EM image processing, 103,180, 55,379, 14,155, 460,806, 70,095, 10,611, and 39,444 particles were selected through 2D- and 3D-classification procedure and used for structure determination of Cx36LMNG-WT, Cx36Nano-WT, Cx36Nano-ΔN8, Cx36LMNG-BRIL, and Cx36Nano-BRIL-ΔN16, respectively. Reducing particle numbers in further 3D-classification negatively affected the resolution and quality of 3D-reconstruction maps.
Data exclusions	For cryo-EM, bad particle images were removed using 2D- and 3D-classification in cryoSPARC and Relion software.
Replication	Prior to data collection cryo-EM image processing, we extensively performed screening. We observed that Cx36-WT constructs top-view preferred orientation, but Cx36-BRIL shows side-view preferred orientation. The reproducibility was confirmed in micrographs and 2D classification using another small data sets.
Randomization	The particle images were automatically selected during particle picking procedure and used for 2D-classification and 3D initial model without symmetry imposition. The resolution of the structures was calculated using gold-standard Fourier shell correlation (FSC).
Blinding	All data collection and image processing procedures were automatically performed in unbiased manner which is generally used in the field of cryo-EM.

Reporting for specific materials, systems and methods

We require information from authors about some types of materials, experimental systems and methods used in many studies. Here, indicate whether each material, system or method listed is relevant to your study. If you are not sure if a list item applies to your research, read the appropriate section before selecting a response.

Materials & experimental systems

n/a	Involved in the study	
☒	☐	Antibodies
☐	☒	Eukaryotic cell lines
☒	☐	Palaeontology and archaeology
☒	☐	Animals and other organisms
☒	☐	Clinical data
☒	☐	Dual use research of concern

Methods

n/a	Involved in the study	
☒	☐	ChIP-seq
☒	☐	Flow cytometry
☒	☐	MRI-based neuroimaging

Eukaryotic cell lines

Policy information about cell lines and Sex and Gender in Research

Human embryonic kidney (HEK) 293E cells (ATCC, CRL10852) and Spodoptera frugiperda (Sf9)cells (ATCC, CRL-1711).

Cell line source(s)	HEK293E and sf9 cells were obtained from American Type Culture Collection (ATCC).
Authentication	HEK293E and sf9 cells were authenticated by morphology and growth characteristics.
Mycoplasma contamination	HEK293E cells were periodically confirmed as negative for mycoplasma contamination.
Commonly misidentified lines (See ICLAC register)	No commonly misidentified cell lines were used in this study.
